# Nociception Level Index-Directed Erector Spinae Plane Block in Open Heart Surgery: A Randomized Controlled Clinical Trial

**DOI:** 10.3390/medicina58101462

**Published:** 2022-10-16

**Authors:** Cosmin Balan, Dana R. Tomescu, Liana Valeanu, Bianca Morosanu, Iulia Stanculea, Antonia Coman, Anca Stoian, Serban I. Bubenek-Turconi

**Affiliations:** 1 Cardiac Anesthesiology and Intensive Care Department I, Prof. Dr. C.C. Iliescu Emergency Institute for Cardiovascular Diseases, 022328 Bucharest, Romania; 2 Anesthesiology and Intensive Care Department, Carol Davila University of Medicine and Pharmacy, 050474 Bucharest, Romania; 3 3rd Department of Anesthesiology and Intensive Care, Fundeni Clinical Institute, 022328 Bucharest, Romania

**Keywords:** erector spinae plane block, cardiac surgery, nociception, NOL index, fast-tracking, opioid-sparing

## Abstract

*Background and Objectives*: The erector spinae plane block (ESPB) is a multimodal opioid-sparing component, providing chest-wall analgesia of variable extent, duration, and intensity. The objective was to examine the ESPB effect on perioperative opioid usage and postoperative rehabilitation when used within a Nociception Level (NOL) index-directed anesthetic protocol. *Materials and Methods*: This prospective, randomized, controlled, open-label study was performed in adult patients undergoing on-pump cardiac surgery in a single tertiary hospital. Eighty-three adult patients who met eligibility criteria were randomly allocated to group 1 (Control, n = 43) and group 2 (ESPB, n = 40) and received general anesthesia with NOL index-directed fentanyl dosing. Preoperatively, group 2 also received bilateral single-shot ultrasound-guided ESPB (1.5 mg/kg/side 0.5% ropivacaine mixed with dexamethasone 8 mg/20 mL). Postoperatively, both groups received intravenous paracetamol (1 g every 6 h). Morphine (0.03 mg/kg) was administered for numeric rating scale (NRS) scores ≥4. *Results*: The median (IQR, 25th–75th percentiles) intraoperative fentanyl and 48 h morphine dose in group 2-to-group 1 were 1.2 (1.1–1.5) vs. 4.5 (3.8–5.5) µg·kg^−1^·h^−1^ (*p* < 0.001) and 22.1 (0–40.4) vs. 60.6 (40–95.7) µg/kg (*p* < 0.001). The median (IQR) time to extubation in group 2-to-group 1 was 90 (60–105) vs. 360 (285–510) min (*p* < 0.001). Two hours after ICU admission, 87.5% of ESPB patients were extubated compared to 0% of controls (*p* < 0.001), and 87.5% were weaned off norepinephrine compared to 46.5% of controls (*p* < 0.001). The median NRS scores at 0, 6, 12, 24, and 48 h after extubation were significantly decreased in group 2. There was no difference in opioid-related adverse events and length of stay. *Conclusions*: NOL index-directed ESPB reduced intraoperative fentanyl by 73.3% and 48 h morphine by 63.5%. It also hastened the extubation and liberation from vasopressor support and improved postoperative analgesia.

## 1. Introduction

Cardiac surgery with sternotomy is associated with significant perioperative nociception of musculoskeletal, visceral, and neurogenic origins [1]. Opioids have been the mainstay of pain management, but their side effects complicate patient recovery and increase costs. Consequently, opioid-sparing has become a fundamental tenet of the enhanced recovery after surgery (ERAS) paradigm, aiming to reduce the length of stay (LOS) and optimize resource utilization [2].

Neuraxial techniques and paravertebral blocks are pivotal ERAS instruments in thoracic surgery, but concerns about bleeding complications have traditionally hindered their adoption in cardiac surgery. Recently, ultrasound-guided chest wall fascial plane blocks have emerged as an efficient analgesic alternative [3]. Technical simplicity and an excellent safety profile in the context of anticoagulation make compelling arguments for their consideration in cardiac surgery [4].

Fascial blocks must match surgical procedures to maximize clinical gains and avert complications [5]. A bilateral erector spinae plane block (ESPB) is well-suited for cardiac surgery with sternotomy. A thoracic ESPB consists of local anesthetic (LA) deposition between the T_5_ transverse process and the erector spinae muscle, resulting in multimetameric somatic and visceral analgesia [6]. Several studies demonstrated that adding bilateral ESPB to general anesthesia could accelerate postoperative rehabilitation and alleviate pain after open heart surgery [7,8,9,10]. However, ESPB does not eliminate but rather variably spares opioid usage depending on the level of proficiency in performing the block and a number of patient-related factors, including parasternal sensory innervation, interfascial plane anatomy, and nervous fiber composition [11]. 

Objective real-time intraoperative nociception monitoring may help prevent overdosing or underdosing opioids and personalize nociception control. The PMD-200^TM^ (Medasense Biometrics Ltd., Ramat Gan, Israel) monitor can track several nociception-related parameters, including heart rate, heart rate variability, pulse wave amplitude, level and fluctuations of galvanic skin response, skin temperature, and movement, to derive the Nociception Level (NOL) index, a real-time measure of the individual’s response to nociception [12]. The NOL index surpassed classical indicators of nociception, such as heart rate and blood pressure [13], and helped reduce postoperative pain, opioid usage, and hypotensive events during general anesthesia [14]. In addition, it remained reliable when general and regional anesthesia were combined [15]. By extension, the NOL index may be applied in cardiac surgery before cardiopulmonary bypass (CPB) initiation to evaluate the block success and individualize opioid administration.

The authors of this randomized controlled trial compared NOL index-directed general anesthesia (NDGA) to NDGA and ESPB combined to see if adult patients undergoing cardiac surgery with sternotomy could benefit from ESPB in terms of reducing perioperative opioid consumption. In addition, metrics of analgesic quality, fast-tracking, vasopressor usage, and adverse events were studied.

## 2. Materials and Methods

### 2.1. Study Design and Patient Enrollment

This prospective, single-center, open-label, randomized controlled trial was performed from December 2019 to May 2021 in adult patients undergoing on-pump cardiac surgery. It was approved by the Institutional Review Board for Biomedical Research of Prof. Dr. C.C. Iliescu Emergency Institute for Cardiovascular Diseases, Bucharest, Romania (2019.07.26/18750) and registered with ClinicalTrials.gov (accessed on 26 July 2019) (Identifier: NCT04338984).

After obtaining written consent, patients that met the following criteria were included in the study: an age range of 18–75 years, elective surgery, and sinus rhythm. The exclusion criteria were as follows: allergy to medications used in the study; body mass index larger than 35; abnormal coagulation profile; emergency or redo surgery; American Society of Anesthesiologists class 4 or higher; pharmacological, or mechanical preoperative cardiocirculatory support; and severe left ventricular dysfunction (i.e., left ventricular ejection fraction less than 30%).

**Figure 1:**
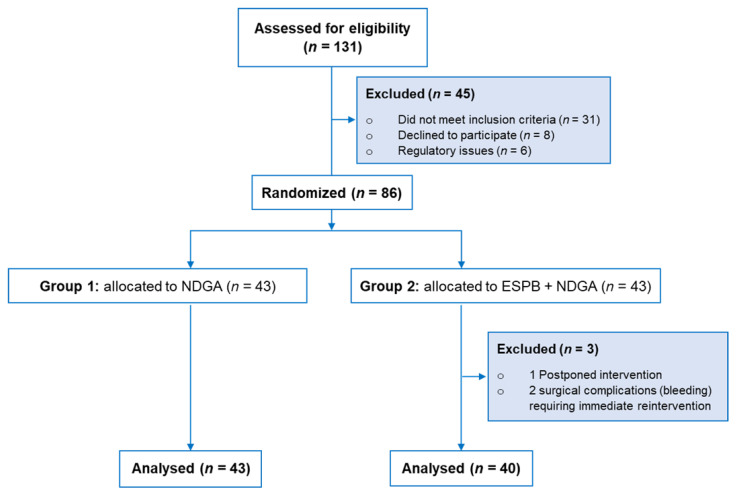
CONSORT diagram showing the patient flow. Abbreviations: CONSORT, Consolidated Standards of Reporting Trials; ESPB, erector spinae plane block; NDGA, Nociception level (NOL) index-directed general anesthesia.

A physician unassociated with the study enrolled the patients. Following enrolment, patients were allocated one-to-one using a random permuted block generator (i.e., block size 4:6:8) into two groups: group 1 (Control) received NDGA, and group 2 (ESPB) received bilateral single-shot US-guided ESPB followed by NDGA (Figure 1).

### 2.2. Anesthesia Management

#### 2.2.1. Preoperative

Patients were explained the 11-point pain intensity numeric rating scale (NRS), where zero indicates no pain and 10 indicates the worst possible pain. Upon arrival to the operating room, oxygen was administered by face mask and standard monitoring was applied, including electrocardiography with automated ST segment analysis, pulse oximetry, and non-invasive blood pressure cuff. After skin asepsis with chlorhexidine 2%, a 16-gauge peripheral intravenous cannula was inserted in the dorsum of the right hand. Skin asepsis was repeated and, under local anesthesia with lidocaine 1%, a 20-gauge left radial arterial cannula was inserted for invasive arterial pressure monitoring. A bispectral index (BIS) sensor was applied to the patients’ foreheads to monitor the depth of anesthesia. The NOL index probe was also attached to all patients on the middle right finger. A period of two minutes devoid of noxious stimuli was ensured to allow calibration of the PMD-200^TM^ monitor.

**Figure 2:**
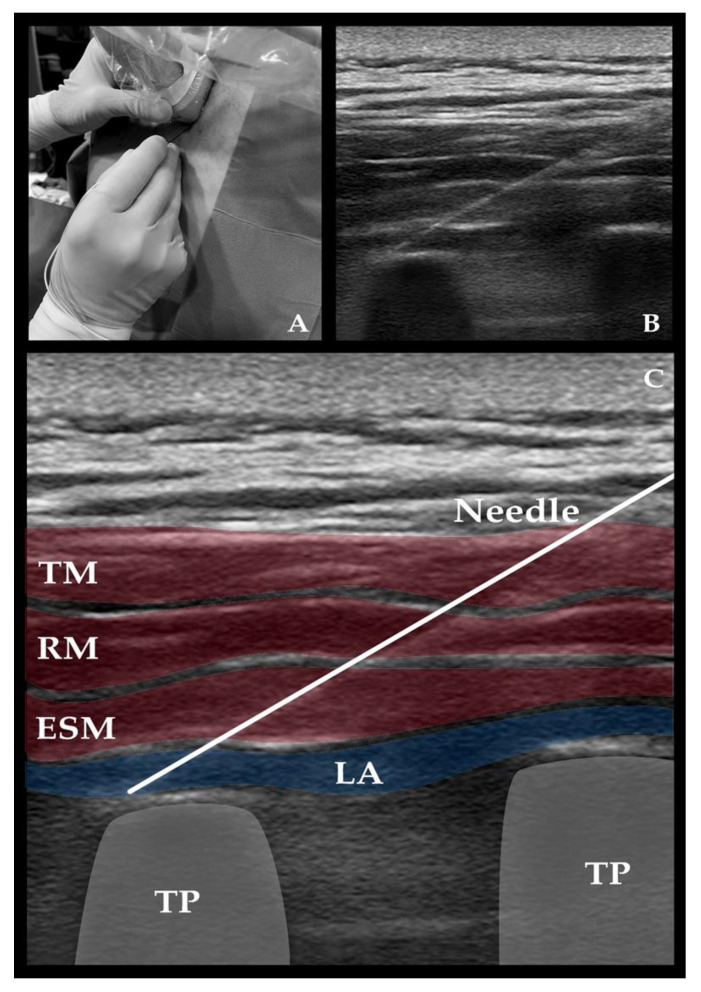
Ultrasound-guided erector spinae plane block. (**A**) clinical anatomy showing parasagittal scanning with a caudal-cranial in-plane approach; (**B**,**C**) sonographic anatomy at the level of the T_5_ transverse process (TP). Local anesthetic (LA) is deposited underneath the erector spinae muscle (ESM), causing an extensive longitudinal separation of the interfascial plane. The transverse processes should appear as flat, squared-off acoustic shadows flanked by an ill-defined pleural line. Abbreviations: RM, rhomboid major muscle; TM, trapezius muscle.

All blocks were performed by a single investigator before induction. Patients from group 2 were placed in the sitting position and provided skin asepsis with chlorhexidine 2% and local anesthesia with lidocaine 1%. To ensure real-time ultrasound guidance, a Phillips CX50 system with a 3–12 MHz L12-3 linear transducer was used (Koninklijke Philips N.V., Eindhoven, The Netherlands). The probe was positioned parasagittal, 2–3 cm lateral from midline, over the T_5_–T_6_ transverse process, identified as a flat, squared-off acoustic shadow flanked by an ill-defined pleural line. A 22-gauge 80 mm needle (SonoPlex II, Pajunk^®^ GmbH Medizintechnologie, Geisingen, Germany) was inserted in-plane, caudocranially, at a 30° angle, across a three-layered muscle bundle (i.e., trapezius, rhomboid major, and erector spinae) until it reached the T_5_–T_6_ transverse process. Correct positioning was certified by hydro-location with saline 2–3 mL, and, after negative aspiration of blood, 1.5 mg/kg 0.5% ropivacaine mixed with dexamethasone 8 mg/20 mL was administered on each side (Figure 2). Classical sensory evaluation of block success (i.e., pinprick and cold stimulation) was waived, given the intraoperative use of the NOL index. Twenty minutes between skin incision and ESPB was always ensured.

Anesthesia induction was performed with propofol 1.5 mg/kg, fentanyl 5 µg/kg, and rocuronium 0.6 mg/kg, followed by protective volume-controlled mechanical ventilation. Afterward, a triple-lumen central venous line, a nasal temperature probe, a urinary catheter, and end-tidal carbon dioxide monitoring were established. The central line was introduced under ultrasound guidance in the right internal jugular vein, using an in-plane approach.

Cefuroxime 1.5 g or vancomycin 15 mg/kg in patients with known beta lactam allergy secured surgical antimicrobial prophylaxis. Stress ulcer prophylaxis was ensured with intravenous omeprazole 40 mg. Both groups received intravenous paracetamol 1 g after induction.

#### 2.2.2. Intraoperative

Before and after CPB, anesthesia was maintained with sevoflurane (1.5–2.5%) in a mixture of oxygen and air (50:50). During CPB, maintenance of anesthesia was achieved with propofol infusion. BIS values of between 40 and 60 were targeted until the end of the surgery. Intermittent doses of rocuronium were given to sustain the neuromuscular blockade.

For both groups, optimum nociception-antinociception (NAN) balance was defined as a NOL index between 10 and 25 on a scale from 0 to 100, where 0 indicates a lack of nociception and 100 indicates extreme nociception. Because the NOL index is, in part, a time-variable photoplethysmographic output, it is inoperative during CPB and may be altered after coming off CPB by cardiac pacing. Hence, NAN balance monitoring was available only until CPB initiation. After induction, fentanyl was started at 2 µg·kg^−1^·h^−1^, and the following rules were applied to achieve NAN balance in both groups: (1) if the NOL index is >25 for more than 60 s, increase the infusion by 0.5 µg·kg^−1^·h^−1^ and bolus 1 µg/kg; (2) if the NOL index is <10 for more than 60 s, decrease the infusion by 0.5 µg·kg^−1^·h^−1^; (3) after a dose change, allow an observation window of three minutes; and (4) stop the fentanyl infusion when it reaches 0.5 µg·kg^−1^·h^−1^ with the NOL index ≤ 25 for more than ten minutes.

From CPB initiation until the end of the surgery, fentanyl administration was conducted in both groups to ensure hemodynamic stability within 15% of the mean arterial blood pressure (MAP) recorded during optimum NAN balance. MAP is highly non-specific; hence, integrating MAP determinants (e.g., vasoactive drugs, CPB flow, anesthetics, temperature fluctuations, and fluids) was endorsed consistently to help differentiate noxious from non-noxious stimuli in the absence of objective NAN monitoring.

Transesophageal echocardiography was standard to monitor and guide the administration of cardiovascular drugs (i.e., norepinephrine, dobutamine) and fluids.

#### 2.2.3. Postoperative

Standard monitoring was resumed after intensive care unit (ICU) admission, and transthoracic echocardiography was applied to guide hemodynamic management.

Extubation was performed when a minimum set of weaning criteria were met: (1) normothermia (temperature > 36 °C); (2) adequate perfusion with dobutamine ≤ 5 µg·kg^−1^·min^−1^; (3) mean arterial pressure ≥60 mmHg; (4) no surgical bleeding and normal coagulation profile; (5) adequate gas exchange defined as an arterial partial pressure of oxygen to fraction of inspired oxygen ratio ≥250 mmHg with a positive end-expiratory pressure < 7 mbar; (6) sustained respiratory effort (tidal volume ≥6 mL/kg with a respiratory rate of 10–20 min) with pressure support ≤ 7 mbar; and (7) wakefulness and adequate cough reflex.

The nursing staff was instructed to administer all patients’ intravenous paracetamol 1 g every 6 h starting with ICU admission. Pain assessment was initiated immediately after extubation. NRS scores with in-bed mobilization were recorded at 2 h intervals for the first 12 h and then at 6 h intervals until 48 h of ICU stay or discharge, whichever came first. Intravenous morphine 0.03 mg/kg was prescribed for NRS scores ≥4 by attending anesthetists.

### 2.3. Outcomes

The primary endpoint was total intraoperative fentanyl consumption. The secondary endpoints were: (1) pre-CPB intraoperative fentanyl consumption (i.e., from induction of anesthesia until CPB initiation); (2) cumulative morphine consumption 48 h after ICU admission; (3) the number of morphine-free patients 48 h after ICU admission; (4) the time to the first dose of morphine; (5) quality of analgesia assessed with NRS scores at 0, 6, 12, 24, and 48 h after extubation and 1 h after drain removal; (6) the time to extubation after ICU admission; (7) the number of extubated patients 2 h after ICU admission; (8) norepinephrine (NE) consumption during the intervention and 12 h after ICU admission; (9) the time to wean off NE after ICU admission; (10) the number of NE-free patients 2 h after ICU admission; and (11) ICU and hospital LOS. ESPB-related complications and the number of ESPB patients requiring fentanyl rescue before CPB initiation were recorded. Additionally, the incidence of opioid-related events (i.e., pruritus, respiratory depression, and postoperative nausea and vomiting, PONV) and postoperative atrial fibrillation (POAF) was dichotomously (i.e., yes/no) assessed in both groups until ICU discharge or 72 h after ICU admission, whichever came first. Respiratory depression was defined as a respiratory rate of fewer than 10 breaths per minute following extubation, regardless of underlying gas exchange parameters.

### 2.4. Sample Size

There is a dearth of literature examining the role of nociception-directed anesthesia in cardiac surgery. One recent study demonstrated decreased intraoperative sufentanil consumption with the pupillary pain index (PPI) [16]. Contrary to PPI, the NOL index is a summative output likely to be disrupted by CPB or cardiac pacing, explaining why the NOL index has only been used postoperatively in cardiac surgery [17]. To the authors’ knowledge, this is the first study to apply the NOL index from the induction of anesthesia until CPB initiation. Hence, the sample size calculation was based on our institutional data. The mean (± standard deviation, SD) intraoperative fentanyl consumption during NDGA was 4.7 (±1.4) µg·kg^−1^·h^−^^1^. The null hypothesis was that ESPB and NDGA combined are not superior to NDGA in total intraoperative fentanyl consumption by more than a superiority margin (δ) of 30%. For a study power of 90%, the sample size was estimated at 23 patients per group (α = 0.05; β = 0.10; δ = 1.4 µg·kg^−1^·h^−1^; Stata/BE 17.0, StataCorp LLC, College Station, TX, USA). To account for potential patient loss to follow-up of 20%, the minimum number of patients required per group was 28.

### 2.5. Statistical Analysis

All statistical analyses were performed using NCSS 2022 Statistical Software (NCSS, v22.0.2, LLC, Kaysville, UT, USA). Visual inspection and the Shapiro–Wilk test were used to check normality for quantitative variables. Quantitative data were expressed as mean (±SD) if normally distributed or median (interquartile range, IQR, 25th–75th percentiles) if non-normally distributed. Categorical variables were presented as absolute numbers and percentages.

For primary outcome analysis, the Mann–Whitney U test for superiority by a margin (δ = 1.4 µg·kg^−1^·h^−^^1^) was applied. Nonparametric quantitative secondary outcomes were evaluated with the Mann–Whitney U test. Binary secondary outcomes and adverse events shared by the two study groups were compared using the chi-squared test or Fisher’s exact test, depending on the number of observations.

The Kaplan–Meier plot was used to analyze time-to-event data (i.e., time to receive morphine and time to extubation) and the Mantel–Cox log-rank test to compare the curves between the two groups of patients. For all comparisons, a two-tailed *p* < 0.05 was considered significant.

## 3. Results

The CONSORT flow diagram shows the patient distribution and allocation (Figure 1). From December 2019 to May 2021, one hundred and thirty-one adult patients scheduled for cardiac surgery with midline sternotomy were assessed for eligibility. Forty-five were excluded at enrolment; eighty-six were randomized to one of the two study groups. In addition, two patients in the ESPB group were excluded due to surgical complications (i.e., bleeding) requiring immediate reintervention after ICU admission, and one was rescheduled. Accordingly, we analyzed 43 patients in group 1 (Control) and 40 in group 2 (ESPB).

There were no differences in demographics, surgery characteristics, surgical procedures, medical history, preoperative risk heart function, and intraoperative monitoring parameters (Table 1).

### 3.1. Primary Outcome

**Figure 3:**
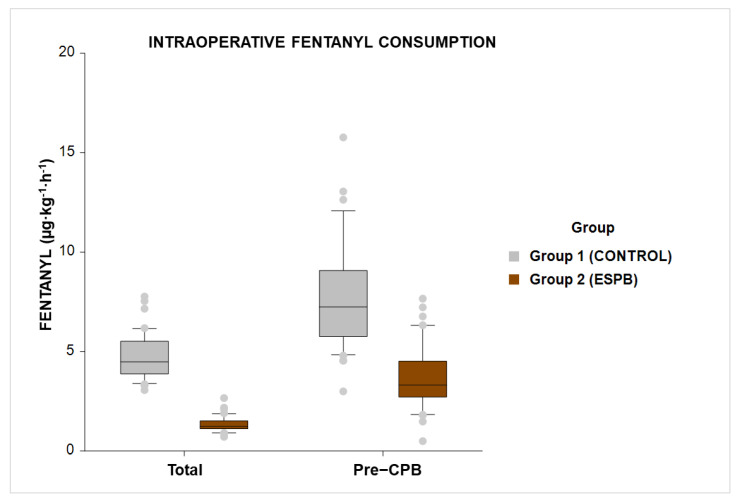
Intergroup comparison for intraoperative fentanyl consumption (µg·kg^−1^·h^−1^). Left, total intraoperative fentanyl consumption (*p* < 0.001). Right, intraoperative fentanyl consumption before cardiopulmonary bypass initiation (pre-CPB) (*p* < 0.001). For the boxplot graph, the horizontal bar indicates the median, the upper and lower box edges indicate the 75th and 25th percentiles, and the upper and lower whisker boundaries indicate the 90th and 10th percentile. Outliers are indicated above and below the whisker boundaries. Abbreviations: ESPB, erector spinae plane block.

Within a NOL index-directed anesthetic protocol, bilateral single-shot US-guided ESPB significantly decreased total intraoperative fentanyl consumption. Patients in the ESPB group received a median (IQR) fentanyl dose of 1.2 (1.1–1.5) vs. 4.5 (3.8–5.5) µg·kg^−1^·h^−1^ in the Control group (*p* < 0.001 for δ = 1.4 µg·kg^−1^·h^−1^) (Figure 3).

### 3.2. Secondary Outcomes

#### 3.2.1. Opioid Consumption

Compared to the Control group, ESPB significantly decreased pre-CPB fentanyl consumption, with a median (IQR) of 3.3 (2.7–4.5) vs. 7.2 (5.7–9) µg·kg^−1^·h^−1^ (*p* < 0.001).

Cumulative morphine consumption 48 h after ICU admission was significantly reduced by the addition of ESPB to NDGA. The median (IQR) doses were 22.1 (0–40.4) vs. 60.6 (40–95.7) µg/kg (*p* < 0.001).

**Figure 4:**
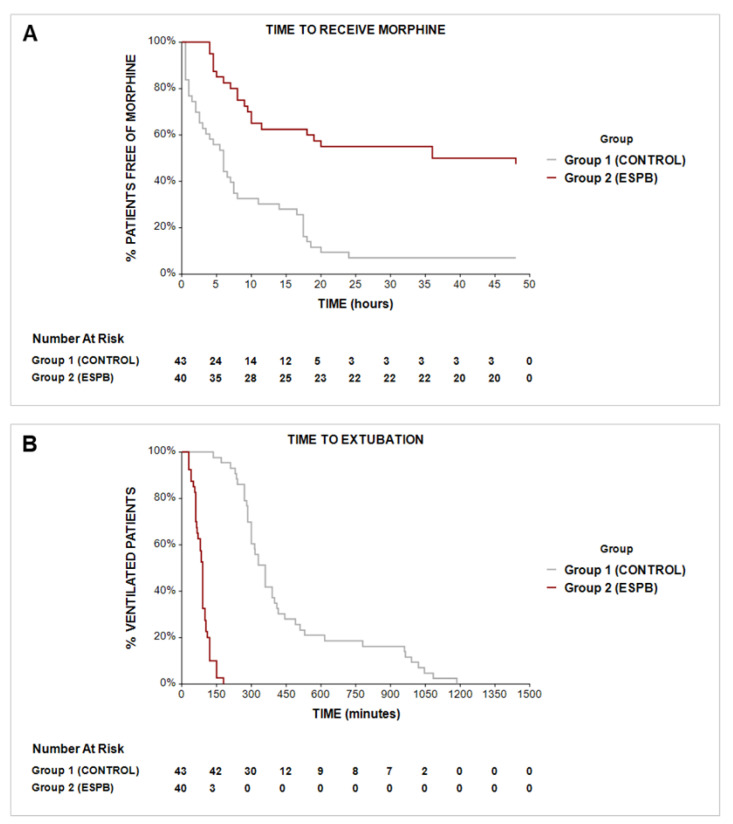
Kaplan–Meier curve to compare the time required to receive morphine after ICU admission (**A**) and the time required to extubate the patient (**B**). The Cox proportional hazard ratio for postoperative morphine in the ESPB-to-Control group was 0.3 (HR: 0.30; 95% CI: 0.18–0.50; *p* < 0.001). The Cox proportional hazard ratio for extubation in the ESPB-to-Control group was 5.24 (HR: 5.24; 95% CI: 2.87–9.57; *p* < 0.001). Abbreviations: ESPB, erector spinae plane block.

Additionally, the number of morphine-free patients 48 h after ICU admission was significantly higher in the interventional group compared to the Control group (47.5% vs. 7%; *p* < 0.001). Additionally, the first time to receive morphine was significantly extended by ESPB, with a median (IQR) from 345 (67.5–795) to 540 (285–1110) minutes (*p* = 0.008). Finally, the Cox proportional hazard ratio for 48 h morphine was 0.3 for the ESPB-to-Control group (HR: 0.30; 95% CI: 0.18–0.50; *p* < 0.001) (Figure 4a).

#### 3.2.2. Quality of Analgesia

NRS scores were compared at six time points: 0, 6, 12, 24, and 48 h after extubation, and 1 h after drain removal. Compared to the Control group, ESPB significantly reduced NRS scores at the first five time-points, with the largest absolute median difference at 6 and 12 h (i.e., the difference in medians was 2) (Table 2). There was no difference in the NRS score one hour after drain removal (*p* = 0.261).

#### 3.2.3. Fast-Tracking

Compared to the Control group, ESPB significantly reduced the duration of mechanical ventilation after ICU admission with a median (IQR) from 360 (285–510) to 90 (60–105) minutes (*p* < 0.001). At 2 h after ICU admission, 87.5% of patients given the block were extubated compared to 0% within the Control group (*p* < 0.001). The Cox proportional hazard ratio for extubation in the ESPB-to-Control group was 5.24 (HR: 5.24; 95% CI: 2.87–9.57; *p* < 0.001) (Figure 4b).

#### 3.2.4. Vasopressor Consumption 

ESPB hastened liberation from vasopressor support after ICU admission. NE consumption, measured from ICU admission until 12 h later, was significantly less in the interventional group compared to the Control group with a median (IQR) of 0 (0–1.3) µg/kg vs. 9 (0–32.3) µg/kg (*p* < 0.001). The time to wean off NE was significantly shorter with than without ESPB, with a median (IQR) of 0 (0–60) vs. 240 (0–720) minutes (*p* < 0.001). Correspondingly, more ESPB patients were free of vasopressor support 2 h after ICU admission (87.5% vs. 46.5%; *p* < 0.001). There was no difference between the groups in total intraoperative NE consumption (*p* = 0.147).

#### 3.2.5. Length of Stay

There were no group differences for ICU LOS (*p* = 0.102) and hospital LOS (*p* = 0.598) (Table 2).

### 3.3. Safety Data

Table 3 reports the incidence of ESPB and opioid-related events (i.e., pruritus, respiratory depression, and PONV) and POAF until ICU discharge or 72 h after ICU admission, whichever came first. The block performance did not result in any complications. For the other events, group differences did not reach statistical significance. Of note, patients given the block experienced none of the opioid’s adverse effects.

### 3.4. Block Effectiveness

Following skin incision, two ESPB patients exhibited a NOL index higher than 25 and required rescue fentanyl according to the intraoperative analgesic protocol. Following sternotomy, one of these and two other patients needed rescue fentanyl, as indicated by the NOL index. Eventually, all three achieved a NOL index of less than 25 before CPB initiation. All the other ESPB patients showed adequate nociception control throughout the incision and sternotomy and did not require rescue fentanyl after induction.

## 4. Discussion

This prospective randomized study demonstrates that the Nociception Level index-directed bilateral single-shot ESPB compared to no block is superior in reducing total intraoperative fentanyl consumption in adult patients undergoing open heart surgery. In addition, ESPB reduced the 48 h morphine and 12 h NE consumption, hastened liberation from vasopressors, enabled fast-tracking, and improved pain scores, but, in this patient population, it did not affect ICU or hospital LOS.

There are several considerations to make concerning the authors’ results. Firstly, this study adds to the growing clinical evidence that bilateral ESPB is an efficient analgesic adjunct for cardiac surgery with midline sternotomy, consistent with the blockade of dorsal and ventral rami of spinal nerves [7,8,9,10,18]. Conversely, Zhang et al. challenged the ventral rami blockade of unilateral ESPB based on cutaneous sensory testing [19]. These two observations, far from being mutually exclusive, may, in fact, coexist. Cutaneous innervation is redundant, with contralateral crossovers and overlaps along the midline, hence the parasternal efficiency of bilateral ESPB with conserved sensation after unilateral block. Equally important, the concepts of differential neural blockade and use-dependent blockade may explain why preoperative sensory testing (i.e., pinprick, cold stimulation) fails to predict the intraoperative magnitude of the antinociceptive effect of ESPB [20].

This leads to the second consideration that continuous objective monitoring of intraoperative NAN balance is fundamentally important. It is the first study to apply the NOL index from the induction of anesthesia to CPB initiation. The pre-CPB period represents a critical window to evaluate the block’s success and tailor opioid administration to its effectiveness. Indeed, it is before CPB initiation that substantial noxious stimuli, including skin incision, sternotomy, and chest retraction, amass and provide a full-scale nociceptive challenge that the block is yet to meet. The NOL index-directed ESPB decreased pre-CPB fentanyl consumption by 54.2%, attesting to the block effectiveness. By the end of the surgery, this difference had reached 73.3%. Previous studies demonstrated the intraoperative opioid-sparing effect of ESPB. Macaire et al., found a 75% reduction in median intraoperative sufentanil [8]. After adjustment for weight and surgery duration, Krishna et al. reported a mean fentanyl usage of 1.15 vs. 5.19 µg·kg^−1^·h^−1^, similar to the authors [7].

Thirdly, postoperative pain scores and morphine consumption likely depend on whether a catheter or a single-shot technique is chosen. In less complex surgeries, single-shot blocks meet two essential ERAS requirements: simplicity and benefit. Compared to catheters, single-shots are less time-consuming, easier to perform, and do not run the risk of displacement. Nevertheless, these advantages come at the cost of a shorter duration of analgesia. Macaire et al. found that catheters can eliminate morphine consumption, improving postoperative rehabilitation and pain scores [8]. In this single-shot study, the addition of dexamethasone to ropivacaine was based on previous reports demonstrating improved and prolonged duration (i.e., beyond 10 h) of postoperative analgesia [21,22,23,24]. The interventional group had a 63.5% decrease in 48 h morphine usage and significantly reduced pain scores up to 48 h after extubation. Two single-shot trials performed in cardiac surgery reported comparable efficiency but only until 6 and 12 h after extubation [7,10]. All this evidence combined merely supports a substantial preemptive effect of single-shot ESPB.

Fourthly, the ESPB group met extubation criteria sooner, achieving a significantly shorter duration of mechanical ventilation. The authors attribute this finding to a fentanyl-sparing effect elicited by ESPB. Other studies that used fentanyl corroborate our findings [7,10]. Conversely, the addition of ESPB to sufentanil-based anesthesia was not associated with faster extubation time [8]. This difference might relate to a variation in opioid choice (i.e., fentanyl vs. sufentanil), fast-tracking protocols, or both. Fentanyl was demonstrated by Ahonen et al. to prolong extubation times compared to sufentanil or alfentanil [25]. Therefore, the fast-tracking effect largely depends on the duration of action of the opioid that the block spares. As a corollary, the fast-tracking effect risks attenuation with shorter-acting opioids such as sufentanil and remifentanil.

Fifthly, in the ESPB group, the authors observed a significant norepinephrine-sparing effect postoperatively but not intraoperatively. This may indicate improved postoperative hemodynamic control in patients with less residual sedation who are closer to being weaned off the ventilator or are already extubated.

Finally, no difference was observed for ICU and hospital LOS. This observation is at variance with Krishna et al. [7] and Statzer et al. [26], who noted a significant reduction in ICU and hospital LOS in patients given the block. Such discrepancies may reflect heterogeneity among centers and even within the same center regarding the endorsement and composition of ERAS pathways.

This study had a few limitations. Firstly, it was a single-centered study that mainly included uncomplicated surgeries, hence the use of a single-shot ESPB approach. Secondly, a sham block design would have been ideal to ensure blinding but was not used since this has remained a controversial issue [27]. Thirdly, the authors did not analyze the LA distribution or classically assess the area of sensory blockade, precluding any correlations of these factors with intraoperative nociception monitoring. Fourthly, given the plethysmographic nature of the NOL index, NAN balance monitoring was limited to the pre-CPB period, preventing any objectivity about the CPB and post-CPB periods. Lastly, long-term follow-up could not be ensured.

## 5. Conclusions

This study demonstrated that, within a NOL index-directed anesthetic protocol, bilateral single-shot ESPB benefits adult patients undergoing open heart surgery in perioperative opioid usage. ESPB reduced the total intraoperative fentanyl and cumulative 48 h morphine requirements by 73.3% and 63.5%, respectively. Other gains included faster extubation and liberation from vasopressor support, and better quality of analgesia up to 48 h after surgery.

## Figures and Tables

**Table 1:** Patient Characteristics and Perioperative Data.

Parameter	Control (*n* = 43)	ESPB (*n* = 40)
Demographics		
Age, years	63 (55–69)	61.5 (52–66)
Gender, male	26 [60.5]	26 [65]
BMI, kg/m^2^	29.6 ± 4.9	28.9 ± 3.9
Weight, kg	82.8 ± 14.1	84.1 ± 13.3
Surgery Characteristics		
Duration of surgery, minutes	296.8 ± 64.1	286 ± 58.4
CPB time, minutes	95 (82–106)	90 (69.2–111.5)
Aortic cross–clamp time, minutes	66 (56–82)	58 (42.5–81.2)
Surgical Procedures		
CABG	19 [44.2]	16 [40]
CABG + valve replacement	3 [7]	4 [10]
AVR	15 [34.9]	9 [22.5]
MVR	1 [2.3]	6 [15]
AVR + MVR	1 [2.3]	0 [0]
ASD repair	0 [0]	2 [5]
VSD repair	0 [0]	1 [2.5]
Myxoma resection	2 [4.6]	1 [2.5]
AAA repair	2 [4.6]	1 [2.5]
Medical History		
Hypertension	41 [95.3]	36 [90]
Diabetes mellitus	8 [18.6]	9 [22.5]
Heart failure	43 [100]	40 [100]
Stroke	4 [9.3]	4 [10]
Chronic kidney disease	4 [9.3]	6 [15]
Endocarditis	0 [0]	3 [7.5]
Myocardial infarction	7 [16.3]	11 [27.5]
Extracardiac arteriopathy	9 [20.9]	15 [37.5]
Chronic opioid therapy	0 [0]	0 [0]
Preoperative Risk and Heart Function		
EuroSCORE II	1.07 (0.76–1.39)	1.02 (0.86–1.56)
LVEF, %	55.0 (50–60)	52.5 (50–60)
Intraoperative Monitoring ^§^		
Heart rate, bpm	84 (75–90)	88 (77.7–90)
Mean arterial pressure, mmHg	67.2 ± 8.1	65.9 ± 7.5
Lactate, mmol/L	1.4 (1.1–1.6)	1.3 (1–1.7)
NOL index > 25	3 [7]	0 [0]
BIS	45 (41–47)	43 (41–48)

Values are mean ± SD, median (IQR, 25–75th percentiles), or number [percentages]. § = measurements were recorded before cardiopulmonary bypass initiation. Abbreviations: AAA, ascending aortic aneurysm; ASD, atrial septal defect; AVR, aortic valve replacement; BIS, Bispectral index; BMI, body mass index; CABG, coronary artery bypass graft; CPB, cardiopulmonary bypass; ESPB, erector spinae plane block; EuroSCORE, European System for Cardiac Operative Risk Evaluation; IQR, interquartile range; LVEF, left ventricular ejection fraction; MVR, mitral valve repair/replacement; NOL index, Nociception Level index; SD, standard deviation; VSD, ventricular septal defect.

**Table 2:** Intergroup comparison for the primary and secondary outcomes.

Outcomes	Control (*n* = 43)	ESPB (*n* = 40)	*p* Value
Primary outcome			
Total intraoperative fentanyl consumption, µg·kg^−1^·h^−1^	4.5 (3.8–5.5)	1.2 (1.1–1.5)	<0.001 ^#^
Secondary outcomes			
Opioid consumption			
Pre–CPB intraoperative fentanyl consumption, µg·kg^−1^·h^−1^	7.2 (5.7–9)	3.3 (2.7–4.5)	<0.001
Morphine consumption 0–48 h, µg/kg	60.6 (40–95.7)	22.1 (0–40.4)	<0.001
Morphine-free patients 48 h after ICU admission	3 [7]	19 [47.5]	<0.001
Time to first dose of morphine, minutes	345 (67.5–795)	540 (285–1110)	0.008
Quality of analgesia ^‡^			
NRS score immediately postextubation	2 (2–4)	1 (0–2)	<0.001
NRS score 6 h postextubation	4 (3–5)	2 (1–3)	<0.001
NRS score 12 h postextubation	4 (3–4)	2 (1–3)	<0.001
NRS score 24 h postextubation	3 (2–4)	2 (0–3)	<0.001
NRS score 48 h postextubation	2 (1–4)	1 (1–2)	0.001
NRS score 1 h after drain removal	2 (1–3)	2 (1–3)	0.261
Fast–tracking			
Time to extubation, minutes	360 (285–510)	90 (60–105)	<0.001
Extubated patients 2 h after ICU admission	0 [0]	35 [87.5]	<0.001
Vasopressor consumption			
Intraoperative NE consumption, µg·kg^−1^·h^−1^	1.1 (0.6–2.2)	1.9 (0.6–3.1)	0.147
NE consumption 0–12 h after ICU admission, µg/kg	9 (0–32.3)	0 (0–1.3)	<0.001
Time to wean off NE after ICU admission, minutes	240 (0–720)	0 (0–60)	<0.001
NE-free patients 2 h after ICU admission	20 [46.5]	35 [87.5]	<0.001
Length of stay			
ICU LOS, days	2 (2–3)	3 (2–4)	0.102
Hospital LOS, days	7 (7–9)	8 (7–9.75)	0.598

Values are median (IQR, 25–75th percentiles), or number [percentages]. # = for the primary outcome, *p* value was determined considering a superiority margin (δ) of 1.4 µg·kg^−1^·h^−1^; ‡ = NRS scores were determined with in-bed mobilization. Abbreviations: ESPB, erector spinae plane block; ICU, intensive care unit; IQR, interquartile range; LOS, length of stay; NE, norepinephrine; NRS, numerical rating scale; pre-CPB, before cardiopulmonary bypass initiation.

**Table 3:** Intergroup comparison for adverse events.

Events	Control (*n* = 43)	ESPB (*n* = 40)	*p* Value
Opioid-related			
Pruritus	1 [2.3]	0 [0]	NS
PONV	3 [7]	0 [0]	NS
Respiratory depression ^#^	1 [2.3]	0 [0]	NS
Arrhythmia			
POAF	4 [9.3]	5 [12.5]	NS
ESPB-related			
Hematoma	N/A	0 [0]	N/A
LAST	N/A	0 [0]	N/A
Pneumothorax	N/A	0 [0]	N/A

Values are number [percentages]. # = Respiratory depression was defined as a respiratory rate of fewer than 10 breaths per minute following extubation, regardless of underlying gas exchange parameters. Abbreviations: ESPB, erector spinae plane block; LAST, local anesthetic systemic toxicity; N/A, not applicable; NS, non-significant; PONV, postoperative nausea and vomiting; POAF, postoperative atrial fibrillation.

## Data Availability

Data used in this study may be provided by the corresponding author upon reasonable request.
